# Suggestions from Dispensary Experience, for the Surgery of General Practice

**Published:** 1886-02-20

**Authors:** Charles W. Dulles

**Affiliations:** Fellow of the Philadelphia Academy of Surgery; Surgeon to the Out-Patient Department of the University Hospital, and of the Presbyterian Hospital, in Philadelphia


### SUGGESTIONS FROM DISPENSARY EXPERIENCE, FOR THE SURGERY OF GENERAL PRACTICE.

Read before the Philadelphia County Medical Society, December 10, 1834.

BY CHARLES W. DULLES, M. D.,

Fellow of the Philadelphia Academy of Surgery; Surgeon to the Out-Patient Department of the University Hospital, and of the Presbyterian Hospital, in Philadelphia.

It has often seemed to me that the experience gained in the many dispensaries of our large cities is not made of as much service to the profession as it might be, and that it would not be amiss if those who have the advantages which these positions afford would occasionally try to put into accessible shape the lessons which they have there learned, and lay them before their brethren for adoption or correction. And, because I have had to learn from experience some things, which it would have been better for my patients if I had found out in some other way, I have thought it might be worth while for me to invite your attention to certain notions in regard, to the kind of surgery which occurs in general practice, which I have gathered during the past ten years, and which, if they are correct, may be helpful to others; if they are incorrect, I shall be glad to have them criticised.

In order to arrange a somewhat desultory subject in as orderly a way as I can, I shall divide it as follows:

1. The examination and diagnosis of surgical lesions.
2. The cleansing of wounds.
3. The control of hemorrhage.
4. The dressing of wounds.
5. Bandaging.
6. Splints.
7. The sling.
8. Constitutional treatment.

### 1. THE EXAMINATION AND DIAGNOSIS OF SURGICAL LESIONS.

I trust I shall not be deemed officious in urging the importance of thoroughness and discernment in making up a diagnosis as to what is the nature of the lesion for which one is consulted by a sufferer In spite of all that is said and written, mistakes are constantly being made, which greater care would have prevented. I have seen fractures treated as contusions, and contusions as fractures, over and over again.

Two little points, in regard to the sinuses of the face, I would like to speak of. One is the well-enough advocated examination of the teeth, by inspection and tapping, to detect a state of abscess in the alveolus; the other I do not remember to have seen recommended. This is, to test a suspected salivary fistula by bringing a drop of the discharge into contact with a drop of the tincture of chloride of iron on a white surface—a piece of white paper will do—when, if the discharge contains saliva, it will give the pink color which indicates the presence of the sulphocyanide of potassium, a normal ingredient of saliva.

### 2. THE CLEANSING OF WOUNDS.

My own experience has led me to the belief that this salutary proceeding is sometimes overdone. When we see a scalp wound, or a laceration of the face, covered with a scab, even though it be not a very handsome one, good surgery does not, I think, require us to take it off, unless the appearance of the neighboring parts indicates that an inflammatory process is going on under it. Nor when a crushed finger is enveloped in a dry covering of blood and machinery grime, need we think our patient's safety depends upon a thorough removal of these. On the contrary, I should say his rapid recovery often depends upon our letting them alone. But scabs that cover pus may always be removed with advantage; and foul secretions or accumulations can only do harm, and must be cleaned out. So the cleansing of wounds is not only an æsthetic, but also a salutary, procedure. As to the method of cleansing, I am a convert to the views of Mr. Sampson Gamgee, who never uses a liquid for cleansing when it is not specially indicated. Careful mopping with dry cotton or lint will do far more than those who have tried it would imagine. Rubbing is rarely called for, but just touching with the cotton or lint. and pressing it down with more or less firmness, as the circumstances require. But, when the case demands it, we must not hesitate to rub firmly, even a little roughly, or to pick off or cut off what sticks tight to the healthy tissues.

In washing of wounds and sores the same fluid should never be used twice. The best way would be to let the water run upon it from a hose, with a regulated force; but almost, if not quite as good as this, is the plan of having one vessel to hold the wash, and another to catch the drippings, and to apply the wash by letting it fall in a steady stream from a clean sponge or a mass of oakum.

### 3. THE CONTROL OF HEMORRHAGE.

An important part of the preparation of a wound for dressing, is the control of hemorrhage—I do not mean the hemorrhage from large vessels, but that from small ones, such as are usually encountered in the surgery of general practitioners. Our colleague, Dr. Roberts, has, I think, wisely deprecated the routine use of styptics, and I quite agree with him that, for almost all small vessels, the pressure of a well-applied dressing will do all that is needed in the way of controlling hemorrhage. Such a dressing may be made of dry lint, bound on with moderate firmness—actual tightness is not called for—and often one will have, in a little while, an imitation of Nature's favorite method of healing, by the formation of a scab, made up of the dried blood and the tissue of the dressing. The essentials for controlling moderate hemorrhage are dry dressings and moderate compression. Pressure alone is sufficient to control the bleeding from scalp wounds, which are sometimes spoken of as if they were troublesome to deal with. It is remarkable, at times, to hear men describe the pains they have been at to ligate an artery of the scalp, in view of the fact that this is never indispensable. A compress and a bandage will occlude any vessel in the scalp, and almost anywhere else; and, if an unruly patient is likely to pull a bandage off, a pin, even a common one, may be thrust under the vessel and brought out again beyond it, so as to hold it as long as any one could wish. If still greater security be desired, it can be had by adding a "figure 8" to this pin.

And here I wish to add a word as to the need for stopping bleeding. I will go a little further than Dr. Roberts in regard to the innocence of hemorrhages which sometimes causes great uneasiness. Many of the hemorrhages are absolutely beneficial. Of course, one need not be foolhardy, but such hemorrhages as come from superficial wounds may be regarded with the greatest equanimity, and no one need get flustrated in trying to stop them.

Bleeding from the wound of the palmar arch can, I believe, almost always be controlled by a pad and bandage.

### 4. DRESSING OF WOUNDS.

*Dry Dressing.*—Nature's method of protecting wounds is by the process of scabbing; and when we reflect upon the successful way in which this operates in all the lower animals, and often in man too, we may wonder that it should be almost a matter of routine to remove scabs in surgical practice. It may gratify our curiosity, it may even aid our study at times, but it is often of no advantage to the patient, to remove from a disfigured face, or a cut head, the crusts which are Nature's reliable antisceptic dressings. From what I have seen, I believe it to be best to leave such crusts undisturbed whenever possible, and if they are objectionable in an æsthetic sense, simply to cover them with something better looking. Further, it may be said that an artificial scab made with lint, or tarletan, or thin muslin, and collodion, forms one of the best dressings for simple incised and not a few lacerated wounds, which have ever been devised.

In hospital practice, I see many cut heads and simple incised wounds even after the removal of tumors, which go to a prompt and uninterrupted healing under the first dressing of this sort. Similarly, scabs may be formed by allowing lint to become saturated with the oozing of a wound exposed to the air. Dry powdered borax, or boric acid, or iodoform, may also be used to promote the formation of a crust. In all these cases, however, it is important to watch lest the crust binds down offensive discharges, as any scab may do; when this happens, the crust must, of course, be removed, and the wound cleaned.

In the case of strumous ulcers and the weak granulations of large burns, I have had the happiest results from setting aside ordinary dressings, and applying a powder in this way. In these latter cases, I have sometimes practised exposure of the granulating surface to the air until the serous film covering them has coagulated and formed a species of skin over them. And, to my astonishment, I have seen such a film actually transformed into thin skin without displacement. This is a fact which, I believe, does not accord with the accepted laboratory idea of new skin formation; but it is a fact, nevertheless.

*Lead Water and Laudanum* is but little better than cold water, so far as my experience would indicate; although it is suited to cases that are especially hot and painful. But, I believe, this ought never to be covered up, as it very often is, with impervious coverings.

*Dilute Alcohol* is another refreshing dressing, if it be allowed to evaporate, and be removed at the first sign of pain.

*Ointments.*—To discuss fully the ointments in use in simple surgery, would require more time than you have to give me. A piece of lint or muslin should be spread with the ointment, and trimmed down to the exact size of the sore. If spread on the adjacent skin, it will often, after a while, set up an artificial eczema, which is very annoying to the patient.

*The Poultice.*—I now come to a subject which has interested me a great deal, and about which I have some convictions, which may be exaggerated, but which are founded on careful observations, made during about five years. One of these convictions is, that the use of poultices is very much overdone. Poultices are of service when it is desired to increase vascular activity in low grades of inflammation, with depressed circulation, and when it is desired to promote or increase pus formation. But I think they do their work in a short time, and that their prolonged use may bring about a condition in which Nature seems unable to get beyond the production of a very feeble and unhealthy sort of tissue. Kept hot and frequently changed, so as to get away the filthy discharges, for a few days they are invaluable; but allowed to cool, left on long at a time, and continued for many days, they do great harm. When a slough is to come away, as after cauterization, or the opening of a felon or carbuncle, nothing which I know of equals a poultice for comfort and effectiveness. But, even in these cases, one should, I think, give them up as soon as the slough is away, and treat the wound as a simple ulcer.

There are no cases which have so much enforced this conviction upon me as those of deep inflammations of the hand and foot—felons and palmar and plantar abscesses. I have myself seen, and so have those who have followed my service in the surgical out-patient departments of the University and Presbyterian Hospitals, many cases which have illustrated the advantages and disadvantages of the use of poultices in the most impressive manner. Only this autumn I have seen three cases where hands affected with deep palmar inflammation have been almost sacrificed to the persistent use of the poultice—all three of them turning immediately back to a recovery as soon as the poultices were laid aside and Nature given a chance to do what she could without them. I may say something similar about felons. I have seen felons well opened and then, too long poulticed, kept unhealed for a long time, the tissues of the finger become boggy and of very low vitality, which recovered promptly when Nature was let alone for awhile, and a little attention paid to the general system.

The result of these observations has been, that I make but little and brief use of poultices in these troubles. A felon I open deeply whenever I think there is pus actually present—never before, for then they can be aborted—then I encourage bleeding by a good soak in very hot water; then I poultice for *one day only*, soaking frequently in water as hot as can be borne. After this I dress with pure laudanum, or lead water and laudanum, or a simple ointment, unless there is obviously a slongh forming; and I usually can dismiss my patient in a few days. When a felon has gone on to destruction of the vitality of bone or tendon, poultices may be used longer; but I believe one should be always on the look-out for the time when they can be thrown aside.

The best treatment of palmar and plantar abscess, or rather of deep inflammations of the hand and foot, cannot be stated in a few words; but alas! for the patient whose doctor is too timid to use the knife, and too assured of the saving grace of the poultice. Too little of the one and too much of the other is a sad combination.

*Strapping with adhesive plaster*, for ulcers, is a troublesome, though very valuable, surgical procedure. It is common in this country to put straps on only part-way round a limb, and to fear the strangulation, which may follow going all the way around.

But this fear is groundless. In England, straps are applied by placing the middle of one at the part opposite the ulcer, carrying the two ends forward, crossing them over the ulcer and fastening them down at the opposite side from which they start; and I have practiced this method myself with perfect safety and success. So much as this is, however, rarely necessary. A good plan is to apply narrow straps *at intervals* over an ulcer and to place on top this interrupted adhesive-plaster support some stimulating ointment on lint—and over all, cotton batting, or oakum, and a bandage. But two things sometimes neglected, are essential to the best success of strapping; one is that the straps be not too wide—say about an inch or less in width—another is, that they should draw the sides of the ulcer together a little, and not simply be plastered against it.

The pressure which can be secured with adhesive straps I have also found useful in a number of inflammatory conditions. I need not mention the strapping of inflamed breasts. But the application of narrow straps will also furnish great relief in the case of boils and carbuncles, and I have had cases of paronychia which resisted assiduous treatment for a long while, but in which immediate relief and rapid recovery followed the application of a circular dressing of adhesive plaster round the end of the finger.

*Collodion.*—This is another agent which may do good service in minor surgery. Many wounds can be easily and effectively coaptated by drawing the edges together, laying over them a strip of tarletan or other bandage, and saturating it with collodion. It should be remembered, however, if one is dealing with children, that collodion applied to a raw surface is very painful for awhile. In applying dressing to the face, we may often dispense entirely with a bandage by using collodion in this way, or by placing against a small wound, or ulcer, or fistulous opening, a little absorbent cotton and gluing its edges down with the collodion, and a neat, soft, absorbent, but impermeable, dressing will be made.

5. BANDAGING.

A mistake is sometimes made in bandaging too tight, and I have once seen a case where gangrene was caused in this way. But, fortunately, the time-honored wood-cut which serves in many works on surgery as a warning against this error furnishes the best information most of us get as to what such a thing is. There is another error, much commoner, and that is bandaging too heavily. I have often seen patients who came with a member firmly bound to a splint, with the laudable object of preventing injurious mobility, but loaded down with successive layers of bandage, till the heat had set up an active inflammation, with the accustomary accompaniments of pain and swelling, which subsided when the lightest possible splint was used and the thinnest possible bandage.

Sometimes it is desired to apply water after a bandage has been put on. In such cases, of course, the bandage should be thin and open-meshed, and put on loosely as is consistent with safety. For this purpose, the cheap unbleached muslins are far better than the fine ones furnished by the instrument makers. Water can also be insinuated under a bandage, if the member has first been wrapped in a layer of absorbent cotton or lint.

The placing of cotton under a bandage has other uses than to facilitate the application of water. One of the most important is, to exert uniform pressure, to prevent swelling, to promote absorption of effusions. One who has not tried it systematically would hardly believe what this sort of compression will accomplish; and I think it might be set down as a rule, that all contusions of joints, and most inflammatory swellings, should be subjected to the equable compression and gentle warmth of dry cotton and a pretty firm bandage. Here again I have found it advantageous to follow the suggestions of Mr. Sampson Gamgee, and have come to prefer this method to the traditional lead water and laudanum.

### 6. SPLINTS.

I have already once or twice incidentally indicated what I think to be an important point in regard to splints, and worthy of more particular mention—I mean their weight. A splint should be no heavier or thicker than is absolutely necessary. The lighter the better, is, I think, a good principle. Let light pasteboard be used when possible, or the very thinnest wood. Nor need their weight and thickness be increased by padding. This is especially true in regard to splints for the arm, where wooden splints are oftenest used. I find it sufficient to wrap a thin wooden splint in waxed paper, to make it perfectly smooth and keep it clean, and to interpose between it and the arm a double strip of lint. These I fasten in place, on the arm, with three or four strips of adhesive plaster, avoiding the seat of fracture or other injury, and covering all in with a light bandage. Then the parts can be examined at any time by simply removing the bandage, without taking off the splint or disturbing the seat of injury. Of course, little wads of cotton may be placed where the member does not touch the splint, and bony prominences must not be pressed too hard against it.

And here I wish to urge upon your attention what I think the best splint for the forearm and hand. Since adopting it, I have found that, like many other supposed discoveries, it is by no means new. But it is so little used that I think it can hardly be much better known to many others than it was several years ago to me; I mean *the posterior, straight splint.* Any one who studies a forearm will see that when the hand and finger are extended, the dorsal surface is almost an accurate plane, while the ventral surface is very uneven. Arguing from this, I thought it well to follow the apparent hint of Nature, and to use this surface for my splints. I soon found that I could treat injuries of the forearm and hand requiring a splint, very successfully with a thin, strainght splint, applied in the way just described. And I may say that I have found it much easier to prevent stiffness of the wrist-joints—the bane of fractures at the lower end of the radius—by this, than by the time-honored Bond's splint, which I have not used for several years. With the Bond's splint I have, in former years, had much trouble from stiffness, and seen much trouble when it was used by others, because, while the position of the hand seems to be favorable to motion, I have not found it really so, but that the patient's fingers are either bound to it too firmly, or they themselves clasp the block so constantly and so rigidly, in spite of all injunctions to the contrary, as to tend to stiffening of all the joints involved. I need scarcely add to what I have already said, any further arguments as to the advantage of the posterior splint in the way of lightness and the facility it affords, when used in the way I suggest, for examining the seat of injury without disturbing it. The Bond's splint, on the other hand, as frequently applied, is heavy, hot, more or less painful, and troublesome to remove for subsequent examination.

### 7. The Sling.

I cannot close these remarks without saying—what my observations lead me to believe is not uncalled for—a word about slings. It ought to be an invariable custom—with those rare exceptions in which for the purpose of drainage it must be reversed—to have a sling so regulated that it will *support the hand at a higher level than the elbow.* A neglect of this very simple, and, I believe, very important, rule is sometimes followed by great pain and swelling of the hand, and a degree of discomfort which would be incredible to one who had not investigated the matter. Further, a sling should be broad enough to support more than a narrow strip of the arm, or one will be apt to find its position marked by a furrow dividing two swollen parts of the arm, in a manner which is not neat, and which suggests possible injury or interference with the most rapid recovery. Another point about the slings concerns the length of time they should be used. Here, again, I think our routine is sometimes too rigid. It cannot be stated exactly how long a sling may be useful; but I have often found it of advantage to let an arm be taken out and allowed to swing at the side, at least occasionally, long before the splint could be dispensed with. If any of you who have not done so already, will try this plan with your patients, I think they will thank you for it, and neither they nor you will regret it.—*The Polyclinic.*

